# Tryptophan Metabolism in Digestive and Extra-Digestive Diseases: Mechanisms, Clinical Implications, and Therapeutic Perspectives

**DOI:** 10.3390/nu18172849

**Published:** 2026-09-01

**Authors:** Ege Tohumcu, Francesca Sofia Puca, Varol Tunali, Lucia Cerrito, Maria Pallozzi, Leonardo Stella, Marta Maestri, Celeste Ambra Murace, Serena Porcari, Simone Varca, Andrea Severino, Antonio Gasbarrini, Gianluca Ianiro, Francesca Romana Ponziani

**Affiliations:** 1 Department of Translational Medicine and Surgery, Catholic University of the Sacred Heart, 00168 Rome, Italy; 2 Clinical Nutrition Unit, Department of Medical and Surgical Sciences, Universitary Policlinic Agostino Gemelli Foundation IRCCS, 00168 Rome, Italy

**Keywords:** tryptophan, kynurenine, 5-HT, Kyn/Trp ratio, gut microbiota, gut–brain-axis

## Abstract

Tryptophan (Trp) metabolism lies at the intersection of nutrition, gut microbiota, mucosal immunology, and systemic inflammation—processes that play key roles in many gastrointestinal and extraintestinal diseases. Trp is an essential amino acid obtained through dietary intake. Beyond its role in protein synthesis, it is metabolized through three principal pathways: the kynurenine pathway, the serotonin/melatonin pathway, and microbial metabolism in the gut leading to indole and related derivatives. The kynurenine pathway represents the primary route of Trp degradation and is strongly linked to inflammatory signaling. The serotonin pathway is particularly important for gastrointestinal physiology, influencing motility, secretion, and visceral sensitivity. In parallel, microbial Trp metabolism produces metabolites that regulate epithelial barrier integrity, modulate mucosal immune responses, and contribute to communication along the gut–organ axes. Across different disease states, several recurring patterns emerge. Inflammatory conditions frequently shift Trp metabolism toward the kynurenine pathway through increased IDO1 or TDO activity, resulting in changes in kynurenine metabolites and in the kynurenine-to-tryptophan (Kyn/Trp) ratio. At the same time, reduced microbial production of indole derivatives may impair aryl hydrocarbon receptor signaling and weaken barrier-protective and immunoregulatory mechanisms. Alterations in the serotonin pathway are also associated with disturbances in gastrointestinal motility and gut–brain communication. Together, these observations highlight Trp metabolism as an important framework for understanding interactions between diet, microbiota, and host responses in health and disease. However, it is important to clarify that much of the currently available evidence remains associative or is derived primarily from preclinical models. This narrative review, based on literature retrieved from major biomedical databases (e.g., PubMed/MEDLINE, Scopus, and Web of Science), aims to provide an updated synthesis of current knowledge on Trp metabolism in disease pathophysiology. Furthermore, while we highlight potential translational applications—such as proposed biomarker development and targeted therapeutic strategies—these perspectives have been moderated to acknowledge the limited level of clinical validation established to date.

## 1. Introduction

Tryptophan (Trp) is an essential amino acid obtained exclusively through diet and serves not only as a substrate for protein synthesis but also as a precursor of multiple bioactive metabolites that connect nutrition, host metabolism, immune regulation, and the gut microbiota [1]. In humans, Trp is routed mainly through the kynurenine pathway, the serotonin/melatonin pathway, and microbial metabolism to indole derivatives. These pathways influence epithelial barrier integrity, mucosal and systemic immunity, gastrointestinal motility, neuroimmune signaling, and host-microbiota crosstalk [1,2,3,4,5]. Their dysregulation has therefore been implicated in a broad spectrum of digestive and extra-digestive diseases—the latter more precisely defined herein as encompassing cardiometabolic, renal, neuropsychiatric, neurodegenerative, and systemic autoimmune and inflammatory disorders. In inflammatory settings, Trp metabolism is frequently redirected toward the kynurenine pathway through increased indoleamine 2,3-dioxygenase (IDO1) or tryptophan 2,3-dioxygenase (TDO) activity, whereas reduced microbial generation of indole derivatives may impair aryl hydrocarbon receptor (AhR)-dependent barrier-protective and immunoregulatory signaling [4,5,6,7,8].

While previously published reviews have largely focused on isolated metabolic branches (e.g., exclusively the kynurenine pathway or microbiota-derived indoles) or single disease categories, the novelty of the present review lies in its integrative, cross-disciplinary approach. In this narrative review, we summarize the major host and microbial pathways of tryptophan metabolism and discuss how their dysregulation contributes to both digestive and systemic diseases, with an emphasis on mechanistic links, clinical implications, and therapeutic perspectives. Rather than attempting an exhaustive, comprehensive catalog of all human pathologies, this manuscript specifically focuses on representative disorders selected due to the robust, rapidly emerging multi-omics and translational evidence linking Trp metabolic shifts to their pathogenesis. By synthesizing these diverse fields, we aim to highlight Trp metabolism as a unifying pathophysiological axis.

## 2. Review Methodology

This review was conducted as a narrative review of the literature on tryptophan metabolism in digestive and extra-digestive diseases. Literature searches were performed in PubMed/MEDLINE, Scopus, and Web of Science using Boolean combinations of the terms ‘tryptophan’, ‘kynurenine’, ‘serotonin’, ‘melatonin’, ‘indole’, ‘microbiota’, ‘aryl hydrocarbon receptor’, and disease-specific terms including ‘inflammatory bowel disease’, ‘irritable bowel syndrome’, ‘celiac disease’, ‘colorectal cancer’, ‘liver disease’, ‘type 2 diabetes’, ‘depression’, ‘Parkinson disease’, ‘Alzheimer disease’, ‘multiple sclerosis’, ‘rheumatoid arthritis’, ‘systemic lupus erythematosus’, and ‘psoriasis’. The primary time interval for the literature search spanned from January 2010 to June 2026, a timeframe chosen to capture the most recent and rapid advancements in metabolomic and microbiome research, though seminal historical papers establishing fundamental biochemical pathways were also retained. The final, updated bibliographic search was performed on 20 June 2026. Reference lists of key articles were also screened for additional relevant publications.

To mitigate selection bias inherent in narrative reviews and enhance the transparency of our evidence selection process, specific criteria guided study inclusion and prioritization. We primarily considered English-language articles and prioritized original studies, systematic reviews, and meta-analyses that were judged by the authors to be most relevant to the mechanistic and translational scope of the review. Specifically, inclusion was restricted to peer-reviewed literature encompassing robust in vitro mechanistic studies, in vivo animal models, human clinical trials, and comprehensive multi-omics analyses. Non-peer-reviewed preprints, conference abstracts lacking full-text availability, and single-case reports without mechanistic data were excluded. Studies were prioritized based on sample size, the strength of their methodological design, and their direct relevance to linking dietary, microbial, or inflammatory drivers to tryptophan metabolism.

Furthermore, given the highly context-dependent nature of host–microbiome interactions, special attention was given to the handling of conflicting evidence. When contradictory findings were identified in the literature—such as the divergent protective versus pathogenic roles reported for specific microbial indole derivatives or variations in kynurenine pathway activation across different disease models—both perspectives were explicitly reported. Rather than excluding contradictory data, we sought to contextualize these discrepancies by discussing potential confounding factors, including differences in experimental models, baseline host microbiome compositions, disease stage, and methodological approaches to metabolomic profiling.

## 3. Tryptophan Biology in Physiological Conditions

### 3.1. Dietary Intake, Absorption, and Systemic Transport

Among the essential amino acids, L-tryptophan is the only proteogenic amino acid that contains an indole ring, a structural feature that underlies many of its biochemical properties [9]. Because humans cannot synthesize Trp, adequate intake depends entirely on diet [10]. In addition to its role in protein synthesis, Trp is the precursor of serotonin, melatonin, kynurenine-pathway metabolites, niacin, and multiple microbiota-derived indole compounds [11]. Dietary sources include meat, fish, eggs, dairy products, soy, legumes, nuts, seeds, and whole grains [9]. Trp requirements vary by age, and both total protein intake and protein source can influence circulating Trp availability; for example, proteins such as α-lactalbumin can increase circulating Trp more effectively than gluten- or zein-rich sources [12,13].

After ingestion, Trp is absorbed predominantly in the small intestine through specialized neutral amino acid transport systems, particularly B0AT1 (SLC6A19), and is subsequently exported to the circulation by basolateral transporters including LAT1, LAT2, and TAT1 [14,15,16]. In plasma, approximately 80–90% of Trp is albumin-bound, whereas the free fraction is available for tissue uptake, including transport across the blood–brain barrier [14]. This balance between intake, intestinal absorption, protein binding, and tissue transport helps determine the substrate available for downstream host and microbial metabolism.

### 3.2. Determinants of Tryptophan Metabolic Routing

Tryptophan metabolism is shaped by several interacting determinants, but their relative importance differs by biological context. All different pathways of tryptophan metabolism are summarized in Table 1. Under physiological conditions, dietary intake, intestinal absorption, and microbiota composition primarily influence substrate availability and the generation of microbial indole derivatives. In contrast, during inflammation, cytokine-driven activation of IDO1 becomes a dominant force that redirects Trp away from serotonin and microbial pathways toward kynurenine production [17,18,19,20]. Stress-related endocrine signaling can further amplify this shift, while physical activity may remodel kynurenine metabolism toward more neuroprotective branches [19,20,21,22,23].

Gut microbiota composition remains a major determinant because bacteria can convert Trp into indole, indole-3-propionic acid, indole-3-lactic acid, and tryptamine, and can also influence cross-feeding networks that shape host exposure to these metabolites [24,25,26,27,28]. Diet interacts strongly with this ecosystem: fiber-rich patterns may favor indole-producer networks, whereas high-fat inflammatory states may enhance kynurenine shunting [29,30,31,32,33]. Finally, host genetics and micronutrient availability, including vitamin B6 status, may further modulate enzyme activity and metabolite balance [17,34]. Overall, altered Trp metabolism should be interpreted as the product of nutritional, microbial, inflammatory, and host-specific factors rather than a single isolated pathway abnormality.

### 3.3. The Serotonin Pathway

Although it accounts for only a small fraction, approximately 1–5%, of total Trp metabolism, the serotonin pathway is of immense physiological significance [35]. The vast majority, about 95%, of the body’s total 5-HT is synthesized within the GI tract [36,37]. This production is carried out primarily by enterochromaffin (EC) cells in the gut mucosa, which use the enzyme tryptophan hydroxylase 1 (TPH1) to convert Trp to 5-hydroxytryptophan (5-HTP), a precursor that is then rapidly decarboxylated to form 5-HT [35]. In the brain, a parallel process occurs in the raphe nuclei neurons, though it is initiated by a distinct enzyme, tryptophan hydroxylase 2 (TPH2) [38]. Once 5-HT is released from EC cells, it acts locally before being rapidly cleared by the serotonin reuptake transporter (SERT), which is expressed on adjacent epithelial cells and, crucially, on circulating platelets. Platelets, which cannot synthesize 5-HT, acquire it from the gut-derived pool via SERT and serve as the main storage reservoir for 5-HT in the periphery [37,39].

In the GI tract, 5-HT functions as an important paracrine signaling molecule as well as a neurotransmitter for the enteric nervous system (ENS). It is the primary regulator of gut motility, initiating peristaltic and secretory reflexes by stimulating intrinsic sensory neurons [38]. It also modulates intestinal secretion of fluid and mucus [37] and plays a key role in visceral sensation. Indeed, dysregulated 5-HT signaling is implicated in the visceral hypersensitivity and abdominal pain characteristic of disorders including irritable bowel syndrome (IBS) [38]. Furthermore, 5-HT is a significant modulator of gut inflammation, exerting both pro-inflammatory and anti-inflammatory effects depending on the specific 5-HT receptors activated, and influencing the severity of conditions such as inflammatory bowel disease (IBD) [37,40]. In the CNS, 5-HT synthesized by raphe neurons acts as a neurotransmitter fundamental to the regulation of mood, appetite, sleep, and cognition [39]. Its dysregulation remains a central feature in the pathophysiology of psychiatric disorders, including major depressive disorder and anxiety disorder [39,41].

### 3.4. The Melatonin Pathway

Melatonin (N-acetyl-5-methoxytryptamine) is synthesized from L-tryptophan (Trp) through a sequential, serotonin-dependent pathway and is produced principally in the pineal gland under strict circadian control [42,43]. The biosynthesis begins with the hydroxylation of tryptophan to 5-hydroxytryptophan (5-HTP) by tryptophan hydroxylase (specifically the TPH1 isoform in pinealocytes), which serves as a major regulatory checkpoint [44]. Subsequent decarboxylation of 5-HTP by aromatic L-amino acid decarboxylase yields serotonin [44,45]. Within the pineal gland, serotonin is converted into melatonin via a tightly regulated two-step enzymatic process: first, acetylation by arylalkylamine N-acetyltransferase (AANAT)—widely acknowledged as the primary rate-limiting enzyme governing the rhythmic nocturnal surge of melatonin—followed by O-methylation of N-acetylserotonin via hydroxyindole O-methyltransferase (ASMT/HIOMT) [45,46].

Beyond its classical chronobiotic role in sleep–wake regulation and photoperiodic synchronization, melatonin also exerts robust antioxidant and cytoprotective effects [42,47]. It acts as a broad-spectrum scavenger by directly neutralizing reactive oxygen and nitrogen species (ROS/RNS) through cascading reactions [47,48]. Furthermore, it reinforces endogenous antioxidant defenses by transcriptionally upregulating protective enzymes such as superoxide dismutase, catalase, and glutathione peroxidase, while mitigating mitochondrial oxidative stress [47,49]. Although the quantitative metabolic flux through the melatonin pathway represents only a minor fraction of total systemic tryptophan utilization compared to the dominant kynurenine pathway, it remains biologically crucial when evaluating tryptophan metabolism in pathological contexts characterized by circadian disruption, excessive oxidative stress, or altered neuroimmune signaling [42,43,47].

### 3.5. The Kynurenine Pathway

The kynurenine pathway is the principal route of Trp catabolism, responsible for degrading over 95% of available Trp [44]. This pathway is initiated by two rate-limiting enzymes: tryptophan 2,3-dioxygenase (TDO), which is primarily active in the liver and regulates systemic Trp levels, and indoleamine (IDO), which is expressed in numerous peripheral tissues and immune cells [45]. IDO is particularly important because it is strongly induced by pro-inflammatory cytokines, most notably interferon-gamma (IFN-gamma) [44,46]. This induction makes the KP a critical link between inflammation and Trp metabolism. The ratio of kynurenine—the central metabolite of the pathway—to Trp is often used as a clinical marker of systemic inflammation. However, it is critical to note that the Kyn/Trp ratio should not be evaluated exclusively as a marker of IDO1 activity, because it may also be significantly influenced by TDO activity, dietary tryptophan availability, and hepatic and renal function, as well as specific analytical conditions. Furthermore, when interpreting these metabolic signatures, biological compartments must be clearly differentiated, since serum, plasma, fecal, tissue, and cerebrospinal-fluid concentrations are not directly interchangeable and often reflect distinct localized processes.

The KP generates several neuroactive and immunomodulatory metabolites. One branch of the pathway produces kynurenic acid (Kyna), a metabolite generally considered neuroprotective. Kyna acts as a broad-spectrum antagonist at ionotropic glutamate receptors, including the N-methyl-D-aspartate (NMDA) receptor, thereby reducing excitotoxicity [45]. However, at high concentrations, its blockade of neurotransmission may contribute to cognitive deficits [46]. In contrast, a separate branch of the pathway causes the production of quinolinic acid (QUIN), a potent neurotoxin [45]. QUIN is an agonist of the NMDA receptor, and its overproduction induces severe excitotoxicity, oxidative stress, and neuronal cell death [46]. This imbalance itself, favoring the neurotoxic QUIN branch, is heavily implicated in the pathogenesis of several neurodegenerative disorders, including Huntington’s disease, Alzheimer’s disease, and the neurological complications of acquired immunodeficiency syndrome (AIDS) [44,45].

Beyond the CNS, the KP is a powerful mechanism of immune modulation. The activation of IDO in immune cells, such as dendritic cells, depletes the local microenvironment of Trp. This tryptophan starvation effectively arrests T-cell proliferation and induces T-cell anergy, a state of immunosuppression. This mechanism is fundamental for maintaining immune tolerance, such as in preventing fetal rejection during pregnancy, but it is also co-opted by cancer cells to evade immune surveillance [44,46]. However, it is crucial to note that KP activation is not a static or uniform feature across all inflammatory conditions [44,46]. Emerging evidence suggests significant variability depending on the stage of the disease (acute flare versus chronic progression), the specific tissue microenvironment, and the patient’s treatment status [44]. For example, while acute inflammatory flares often dramatically spike the Kyn/Trp ratio, chronic low-grade inflammation or prolonged immunosuppressive therapy may present with more subtle, divergent, or even compensatory metabolic shifts [46]. Recognizing these nuances is essential for avoiding oversimplified interpretations of KP metabolites as universal biomarkers, as their diagnostic and prognostic value heavily depends on the clinical context [44,46].

**Figure 1:**
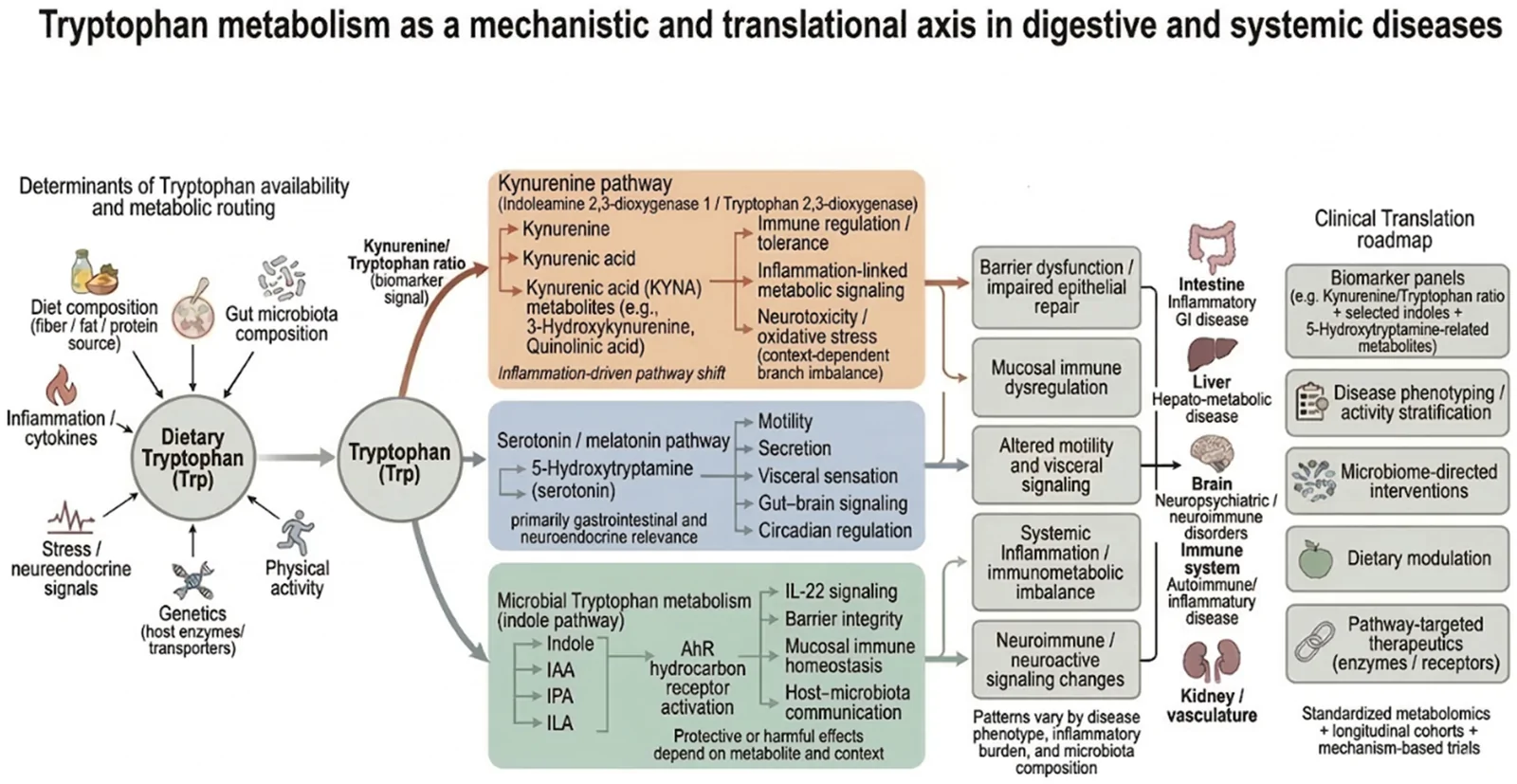
The figure delineates the progression from upstream metabolic determinants to downstream clinical applications. Systemic tryptophan availability is dictated by a convergence of exogenous and endogenous factors, including dietary composition, gut microbiota architecture, systemic inflammation, host genetics, and neuroendocrine signaling. Available tryptophan is subsequently partitioned across three principal metabolic cascades: the host-driven kynurenine pathway, the serotonin/melatonin pathway, and the microbial indole pathway. Dysregulation within these intersecting pathways precipitates specific pathophysiological mechanisms—such as compromised epithelial barrier integrity, mucosal immune dysregulation, and aberrant neuroimmune signaling—which collectively drive the pathogenesis of various digestive and systemic diseases. Ultimately, this conceptual framework provides a roadmap for clinical translation, emphasizing the utility of integrated biomarker panels (e.g., Kyn/Trp ratio alongside selected indoles) for disease phenotyping and the development of targeted therapeutic strategies, including precision dietary modulation, microbiome-directed interventions, and pathway-specific pharmacological targeting. IAA: Indole-3-acetic acid, IPA: Indole-3-propionic acid, ILA: Indole-3-lactic acid.

Figure 1 summarizes the principal host and microbial pathways of tryptophan metabolism, including key enzymes, major metabolites, principal tissue sources, physiological functions, and representative disease associations.

## 4. Tryptophan Metabolism in Digestive Disorders

Table 2 summarizes the main digestive disorders associated with altered tryptophan metabolism, highlighting dominant pathway alterations, key metabolites or markers, evidence type, and putative clinical implications.

### 4.1. Inflammatory Bowel Disease

Inflammatory bowel disease (IBD), including Crohn’s disease (CD) and ulcerative colitis (UC), is a group of chronic, relapsing gastrointestinal conditions characterized by complex interactions among genetic susceptibility, environmental factors, microbial dysbiosis, and impaired intestinal barrier function, leading to dysregulated immune responses and mucosal inflammation [48]. There is increasing evidence that alterations in tryptophan metabolism are a consistent feature of IBD. A meta-analysis by Wang et al. [49] integrating transcriptomic and metabolomic datasets demonstrated that in patients with IBD, there is a reduced expression of the Trp transporters B0AT1 and TAT1 that is associated with lower circulating and higher fecal Trp levels [49]. However, these findings underscore a critical nuance regarding metabolic variability: KP alterations in IBD are highly dependent on disease stage and treatment status [49]. While patients with active UC and CD show significantly higher expression levels of IDO1 and kynurenine 3-monooxygenase (KMO), alongside elevated levels of the metabolite Kyna—resulting in an elevated Kyn/Trp ratio—these metabolic signatures frequently normalize or diverge significantly during clinical remission or following successful targeted biologic therapy [49,50]. This variability indicates that the Kyn/Trp ratio serves as a highly dynamic marker of real-time inflammatory burden and mucosal healing rather than a fixed, static trait of IBD [49,50].

While these human data establish a strong clinical association, mechanistic insights often rely on murine models, which must be interpreted with caution due to inherent differences in baseline microbial ecology and immune system architecture between species [49]. For instance, the study by Zhao et al. examined the effects of dextran sodium sulfate (DSS)-induced colitis on Trp metabolism in mice [51]. Although DSS models are valuable for studying acute epithelial injury, they do not fully replicate the chronic, relapsing immune context of human IBD. In this model, they showed increased serum Kyn levels and a significant decrease in serum Trp and Kyna, mediated by the rate-limiting enzymes IDO-1 and KAT2 [51]. In addition to host enzymatic activity, in fact, the gut microbiota is a key regulator of Trp metabolism. Recent metagenomic studies have shown that patients with IBD exhibit profound alterations in microbiota composition compared with healthy controls. According to Zheng et al., the microbiota of patients with IBD is characterized by reduced Firmicutes and increased Proteobacteria. In particular, in CD, certain bacterial species with anti-inflammatory properties are depleted, whereas E. coli and B. Fragilis are enriched [52]. These compositional changes carry direct implications for tryptophan metabolism because reduced beneficial bacteria decrease the production of indole derivatives, such as IPA and IAA, which normally act as ligands for the AhR, thereby maintaining epithelial barrier integrity and regulating mucosal immune responses [53].

As investigated by Whang et al., ILA produced by lactobacilli also functions as a cross-feeding metabolite, supporting the growth of other indole-producing microbes and consequently sustaining a protective metabolic network [54]. Dietary L-Trp consumption also increases the number of G protein-coupled receptor 15+ (GPR15) colonic regulatory T (Treg) cells in large intestine lamina propria (LILP), influencing the availability of specific AhR ligands responsible for GPR15 expression. Typically, the increase in Treg cells in the LILP is correlated with a decrease in local inflammatory T cells, such as IFNγ- and IFNγ-/IL-17A-producing CD4+ T cells and IL-17A-producing γδ T cells in the LILP, decreasing the risk of developing colitis [55]. Restoring the balance of tryptophan metabolites may be a promising therapeutic strategy for maintaining mucosal homeostasis in IBD. Michaudel et al. (2023) demonstrated that the severity of intestinal inflammation correlates with altered levels of key Trp-derived metabolites xanthurenic acid (XANA) and Kyna [50]. In both human patients and murine colitis models, reduced levels of these metabolites were associated with more severe disease [50]. Supplementation with XANA or Kyna mitigated colitis severity by acting on intestinal epithelial cells (IEC) and CD4+ T cells, largely through AhR activation, promoting IEC proliferation, supporting tissue integrity and healing processes.

### 4.2. Irritable Bowel Syndrome

Irritable bowel syndrome (IBS) is a functional disorder characterized by multifactorial pathophysiology, including disorders of gut–brain interaction, visceral hypersensitivity, gastrointestinal dysmotility, and an imbalance in gut microbiota [56]. Growing evidence suggests that alterations in tryptophan metabolism are associated with gastrointestinal symptoms. Chojnacki et al. investigated alterations in tryptophan metabolism in patients with predominant diarrhea (IBS-D) compared with those with predominant constipation (IBS-C) by measuring urine concentrations of tryptophan metabolites. The results showed that the diarrheic subtype is associated with increased serotonin pathway activity, while constipation correlates with a majorly active kynurenine pathway [57]. According to this evidence, in IBS-M, urinary levels of 5-hydroxyindoleacetic acid (5-HIAA) and serotonin levels in serum were higher during diarrhea periods [58]. Therefore, modulation of dietary Trp intake, but not extreme restriction considering the beneficial effects of some indole derivatives and neuroactive compounds, can lead to reduction in IBS symptoms [59].

These alterations in tryptophan metabolism are closely linked to the composition and function of the gut microbiota. In a longitudinal multi-omics study, Mars et al. analyzed stool samples and colonic biopsies from patients with IBS and healthy controls [60]. They found a significantly higher abundance of multiple Streptococcus spp. individually in IBS-C and IBS-D compared to healthy controls, and a higher relative abundance of lactobacilli in severe IBS-D compared to mild-to-moderate IBS-D. Of particular interest is tryptamine, a microbial-derived metabolite of Trp that activates epithelial 5-HT4 receptors, thereupon enhancing fluid secretion and accelerating intestinal transit [28]. Consistently, patients with IBS-D displayed significantly higher fecal levels of both Trp and tryptamine, which were associated with looser stools and increased bowel frequency.

Trp alterations are associated not only with gastrointestinal symptoms but also with psychological comorbidities, such as anxiety and depression. Han et al. analyzed fecal/serum metabolites and gut microbiota composition of patients with IBS and depression, finding an association among elevated levels of metabolites, including histamine, tryptamine, and kynurenine, and specific bacteria such as Roseburia inulinivorans and Clostridium nexile [61]. They also compared the microbiome of patients with IBS and healthy controls, finding differentially abundant species; 23 were elevated in IBS relative to controls, including Ruminococcus gnavus, Escherichia coli, and Bacteroides plebeius [61]. In particular, they found an increase in several Gram-negative bacteria, including Bacteroides faecis, Escherichia coli, and Klebsiella pneumoniae, in patients with both IBS-D and IBS-C [61]. In addition, Martin et al. showed that psychological comorbidities in patients with IBS improved after administration of the probiotic Bifidobacterium longum NCC300, due to the increase in specific plasma metabolites, including L-tryptophan and N-acetylated-tryptophan [62].

### 4.3. Celiac Disease

Celiac disease (CeD) is an autoimmune enteropathy triggered by dietary gluten, characterized by villous atrophy, mucosal inflammation and dysregulated immune responses. Immunopathogenesis entails complex interactions between gluten-specific T cells, antigen-presenting cells and pro-inflammatory cytokines, eventually resulting in mucosal injury and systemic immune dysregulation [63]. Trp and its metabolic derivatives through kynurenine and indole pathways are emerging as an important factor in the pathogenesis and regulation of CeD. Treated adult and children patients with CeD exhibit decreased plasma levels of Trp and low Trp levels in CeD has also been associated with extra-intestinal symptoms such as poor sleep [64,65]. Torres et al. demonstrated that IDO is highly expressed in inflammatory mononuclear and dendritic cells in the small intestinal mucosa of CeD patients and found a higher Kyn/Trp ratio and a lower Trp level in CeD patients compared to healthy individuals [66]. These metabolic changes were associated with increased tumor growth factor beta (TGF-β), and IL-10 expression in the lamina propria, suggesting that IDO-expressing cells may have a role in mucosal immune regulation and inflammation [66].

Asgari F et al. investigated the role of Trp combined with retinol (Ret) in dendritic cells (DCs) stimulated with gliadine of celiac patients. The results showed that this combination increased the secretion of anti-inflammatory cytokines TGF-β and IL-10 in mature DCs, while inhibiting the production of TNF-α and IL-12. Trp + Ret showed anti-inflammatory effects also by downregulating nuclear factor kappa-light-chain-enhancer of activated B cells (NF-κB) signaling while upregulating retinaldehyde dehydrogenase 2 (RALDH2), IDO, and Programmed Death-Ligand 1(PD-L1) expression, enzymes associated with immune-tolerance [67]. A Trp-rich diet reshapes gut microbiota composition favoring the growth of bacteria belonging to Firmicutes able to metabolize Trp into AhR ligands, like lactobacilli, at the expense of bacteria from the Proteobacteria and Verrucomicrobia, resulting in an increased concentration of AhR agonists and lower kynurenine levels. Higher levels of AhR ligands improved villus-to-crypt ratios, lower intraepithelial lymphocyte (IEL) counts, intestinal paracellular permeability, and duodenal IL-6 expression. Additionally, the administration of AhR ligand producers L. reuteri, and pharmacological stimulation using a direct ligand of AhR like 6-formylindolo carbazole (Ficz), showed the same effects [68]. These pieces of evidence suggest that Trp supplementation, microbial AhR ligand restoration and modulation of Trp pathways may have possible benefits with gluten-free diet in celiac disease patients.

### 4.4. Colorectal Cancer

The metabolism of tryptophan has developed as a critical checkpoint in tumor progression, particularly in colorectal cancer (CRC) [69]. The primary mechanism includes the KP, which catabolizes the majority of dietary Trp. In the tumor microenvironment (TME) of CRC, the rate-limiting enzyme IDO1 is frequently overexpressed [69]. This upregulation of IDO1 has two profound, pro-tumorigenic effects. First, IDO1-mediated Trp catabolism locally depletes Trp, an essential amino acid. This “starvation” state is detrimental to proliferating anti-tumor immune cells, especially effector T-cells, leading to their anergy and apoptosis [69].

Second, the pathway produces a cascade of bioactive metabolites, collectively known as kynurenines [69]. Kyn itself has been identified as an “oncometabolite” that promotes tumor cell survival and invasion. Furthermore, Kyn and other downstream metabolites activate the AhR, a transcription factor that drives a pro-tumor, immunotolerant TME. This includes promoting the differentiation of immunosuppressive Tregs and the polarization of M2 macrophages, which further shield the tumor from immune attack [69,70]. Consequently, enhanced Trp metabolism via the KP is strongly correlated with poor patient prognosis, advanced tumor stage, and immune escape in CRC [70]. This has made IDO1 and other components of the KP prominent targets for novel cancer immunotherapies.

### 4.5. Gastroparesis

The role of tryptophan in gastroparesis is inherently connected to its function as the sole precursor for the neurotransmitter serotonin (5-hydroxytryptamine, 5-HT) [71]. Over 90% of the body’s 5-HT is synthesized in the gastrointestinal tract by enterochromaffin cells, a process initiated by the rate-limiting enzyme tryptophan hydroxylase 1 (TPH1). Serotonin is arguably the most important signaling molecule for regulating gastrointestinal motility. It initiates peristaltic and secretory reflexes, which play a vital role in the coordinated contractions that lead to gastric emptying [72]. Specifically, 5-HT released from EC cells acts on various receptors, including 5-HT4 receptors on enteric neurons, to stimulate prokinetic pathways and promote movement [72].

In the context of gastroparesis, which is defined by delayed gastric emptying, dysregulation of this pathway is a key pathogenic mechanism. Studies have directly shown that serotonin deficiency is associated with delayed gastric emptying [71]. This link is further supported by clinical evidence, as prokinetic drugs used to treat gastroparesis are often 5-HT4 receptor agonists that mimic the effects of serotonin, which has pro-motility effects [72]. Moreover, experimental studies in healthy volunteers have demonstrated that acute tryptophan depletion via a Trp-deficient amino acid drink effectively reduces 5-HT availability and significantly slows gastric emptying, thereby mimicking a gastroparetic state [73]. Therefore, any factor that disrupts Trp availability, its conversion to 5-HT by TPH1, or 5-HT receptor signaling can contribute to the hypomotility seen in gastroparesis.

### 4.6. Small Intestinal Bacterial Overgrowth

Tryptophan metabolism is a key interface between the host and the gut microbiota [74]. In a healthy state, Trp is absorbed in the small intestine, but in conditions like small intestinal bacterial overgrowth (SIBO), this process is disrupted. In SIBO, an excessive bacterial load in the small intestine leads to premature fermentation and metabolism of nutrients, including tryptophan. Thus, not only do bacteria directly compete with the host for Trp, which can reduce its absorption and availability for host processes like 5-HT synthesis, but, as emerging evidence suggests, this altered Trp metabolism plays a direct role in SIBO pathophysiology. A 2021 pilot study specifically investigated the serotonin pathway of Trp metabolism in hydrogen-positive SIBO patients [74,75]. The study found that dysregulation in this pathway may influence the clinical presentation of SIBO, particularly in differentiating between diarrhea-predominant and constipation-predominant subtypes. The authors proposed that urinary 5-hydroxyindoleacetic acid could serve as a non-invasive marker for these metabolic disturbances in SIBO [74].

## 5. Tryptophan Metabolism in Cardiometabolic and Renal Disorders

Table 3 summarizes the principal extra-digestive and systemic disorders associated with altered tryptophan metabolism, including dominant pathway alterations, key metabolites or markers, evidence type, and putative clinical implications. Beyond the gastrointestinal tract, Trp metabolism is increasingly recognized as a regulator of metabolic and cardiovascular homeostasis. In these conditions, low-grade inflammation, microbial metabolite imbalance, and altered host signaling frequently converge on kynurenine and indole pathways, linking diet and microbiota composition to systemic metabolic risk.

### 5.1. Liver Diseases: MASLD, MASH, Cirrhosis, and Hepatic Encephalopathy

The gut–liver axis is central to the pathogenesis of liver diseases, and tryptophan metabolism is a critical signaling pathway within this axis. Dysregulation of Trp metabolism has been implicated in the entire spectrum of liver disease, from metabolic dysfunction-associated steatotic liver disease (MASLD) to cirrhosis and hepatic encephalopathy [76,77,78]. The mechanisms are twofold, involving both the serotonin and kynurenine pathways. Research has shown that a high-fat diet can increase gut-derived 5-HT. This peripheral serotonin, produced from Trp, travels via the portal vein to the liver where it promotes hepatic steatosis (fatty liver) by acting on liver serotonin receptors like HTR2A. In preclinical models, inhibiting this gut-derived serotonin synthesis was shown to ameliorate hepatic steatosis, identifying it as a pathogenic driver of MASLD [76].

As MASLD progresses to metabolic associated steatohepatitis (MASH), chronic inflammation upregulates the IDO1 enzyme. This shifts Trp metabolism away from the 5-HT pathway and towards the KP, similar to what is seen in cancer [77]. An elevated Kyn/Trp ratio is a known marker of systemic inflammation and is associated with the severity of MASH and liver fibrosis [77]. Finally, in end-stage liver disease (cirrhosis), the liver fails to clear metabolites. Abnormal Trp metabolism is strongly implicated in the development of hepatic encephalopathy (HE), a state of neurocognitive dysfunction, as neurotoxic KP metabolites like quinolinic acid and other Trp derivatives can cross the blood–brain barrier and contribute to the neurological symptoms [78].

### 5.2. Type 2 Diabetes Mellitus

Alterations in Trp metabolism, involving both host and microbial pathways, are strongly implicated in the pathogenesis of type 2 diabetes (T2D) and insulin resistance [79]. The KP is frequently upregulated in chronic inflammatory states, including obesity and T2D. This leads to an increased Kyn/Trp ratio, which is often utilized as a marker of IDO enzyme activity, though it must be interpreted cautiously alongside factors such as TDO activity, baseline dietary tryptophan availability, and renal or hepatic function. Evidence from large-scale human cohort studies is robust; a study involving more than 9000 participants found that elevated circulating levels of several KP metabolites, including Kyn, Kina, xanthurenic acid (XANA), and quinolinate, were all positively associated with a higher risk of developing incident T2D [79]. XANA, in particular, has been investigated for its likelihood to impair insulin signaling and promote beta-cell dysfunction [80].

Concurrently, metabolites from the indole pathway show a distinct and often opposing relationship with the disease. The same large cohort study found that indolepropionate, a metabolite produced exclusively by gut bacteria from Trp, was inversely associated with T2D risk [79]. This suggests a protective role. Indolepropionate is regarded as enhancing gut barrier integrity and improving glucose metabolism by promoting glucagon-like peptide-1 (GLP-1) secretion from intestinal L-cells, thus linking microbial Trp metabolism directly to host glycemic control [79,81].

### 5.3. Cardiovascular Disease

Mounting evidence identifies dysregulated Trp metabolism as a novel risk factor for cardiovascular disease (CVD). The link is strongly centered on the inflammatory KP. Data from an EPIC-Norfolk cohort study demonstrated that while higher levels of Trp itself were inversely associated with fatal CVD, a high Kyn/Trp ratio was associated with a significantly higher incidence of CVD [82]. This activation of the IDO enzyme and subsequent production of Kyn metabolites contribute to a pro-inflammatory vascular state, promoting endothelial dysfunction and atherosclerotic plaque development [82,83].

Microbial metabolites also play a dual role in cardiac health. The uremic toxin indoxyl sulfate, produced from bacterial metabolism of Trp in the gut and subsequent sulfation in the liver, is a well-known pro-inflammatory molecule that promotes vascular inflammation and oxidative stress [83]. In contrast, the same microbial metabolite found to be protective in T2D and MASLD, IPA, has been shown to have protective vascular roles [83]. This positions the balance between different Trp metabolic pathways as a key determinant of cardiovascular risk.

### 5.4. Chronic Kidney Disease

In chronic kidney disease (CKD), the decline in renal function leads to the retention of metabolic waste products, many of which are biologically active and toxic. Trp metabolites are the most abundant ones among these uremic toxins [84]. As kidney function deteriorates, metabolites from both the host and microbial pathways accumulate. Metabolites of the host-driven KP are significantly altered from the early stages of CKD and continue to rise through its progression to end-stage renal disease. This accumulation is not simply a marker of declining filtration but actively contributes to the systemic inflammation and significant vascular complications associated with uremia [84].

Simultaneously, the microbial pathway produces classic protein-bound uremic toxins. Gut bacteria metabolize Trp to indole, which is absorbed and converted by the liver into indoxyl sulfate. In a healthy individual, indoxyl sulfate is efficiently cleared by the kidneys. In CKD, its levels rise dramatically. Indoxyl sulfate is highly toxic, directly promoting the progression of renal fibrosis, inducing endothelial dysfunction, and being a major contributor to the high cardiovascular mortality seen in CKD patients [83,85]. These indolic metabolites are known to accumulate in uremic serum, where they compete with other substances for binding to albumin, additionally complicating the uremic state [85].

## 6. Tryptophan Metabolism in Neuropsychiatric and Neurodegenerative Disorders

Trp metabolism is closely linked to the gut–brain axis and has been implicated in several neurological and psychiatric disorders through effects on neurotransmission, neuroinflammation, oxidative stress, and microbiota–host signaling.

### 6.1. Depression and Anxiety

Anxiety and depression are the two most prevalent psychiatric disorders worldwide, often co-occurring and sharing similar chemical pathways. The majority of data indicate that these conditions frequently present together and act as bidirectional risk factors for one another over time. Individuals with anxiety disorders face a significantly elevated risk of developing subsequent depression, while those with depression are similarly predisposed to anxiety [86]. Emerging evidence highlights the central role of aberrant KP activity in both major depressive disorder (MDD) and anxiety. The KP can produce both neuroprotective metabolites, such as Kyna and NAD+ [87]. Multi-omics and metabolomic analyses revealed that L-tryptophan and Kyna were consistently downregulated in patients with MDD, regardless of antidepressant exposure [88]. Increased Kyn levels and Kyn/Trp ratios have been correlated with cognitive dysfunction, such as impaired working memory, in patients with MDD [89]. A higher 3-hydroxykynurenine/Kyna ratio has been proposed as a sensitive biomarker for the early detection of MDD in adolescents [90]. Genetic data support this link, showing that specific single nucleotide polymorphisms within genes involved in the kynurenine pathway (such as IDO1, IDO2, KMO, and KAT1) are associated with a higher risk of depression [91]. Parallel findings in anxiety suggest overlapping mechanisms. Anxiety-like behaviors induced in mice after prolonged chronic restraint stress were accompanied by an increase in 3-hydroxykynurenine and a decrease in Kyna, while 5-hydroxytryptamine was significantly decreased. These outcomes suggest that these metabolites could be potential biomarkers for anxiety and depression [92].

Recent studies have shown that altered Trp metabolism in MDD and anxiety is linked to gut microbiota dysbiosis. Zouth et al. found that the relative abundance of Actinobacteria, Proteobacteria, and Verrucomicrobia phyla increased in adolescents with depression compared to healthy controls (HC). Lower abundance of Firmicutes, Faecalibacterium, Roseburia, Collinsella, Blautia, and Phascolarctobacterium was restored after sertraline treatment.

To establish causality, researchers frequently utilize animal models; however, translating these behavioral and microbial findings to humans requires acknowledging the distinct differences in rodent versus human neurobiology and gut microbiomes. In preclinical models, fecal microbiota transplantation from healthy human patients to mice improved depression-like behaviors and decreased the levels of Kyn and its downstream neurotoxic metabolites [93]. Colonization with Roseburia intestinalis specifically resulted in remarkably increased 5-HT levels, improved gut barrier integrity, and decreased transport of Kyn from the periphery to the brain, improving synaptic plasticity and glial activity [93]. Furthermore, Wang et al. demonstrated that the beneficial effect of indole derivatives act through activation of aryl hydrocarbon receptor-dependent signaling, and a high-tryptophan diet or administering indole derivatives significantly alleviated depression and anxiety-like behaviors in methamphetamine-withdrawal mice [94].

Several other studies have shown the potential beneficial role of certain bacterial taxa in mental disorders through tryptophan metabolism modulation. Pan et al. demonstrated that co-administration of Akkermansia muciniphila and lactic acid increased tryptophan hydroxylase-1 expression and decreased IDO expression, shifting tryptophan metabolism towards the production of 5HT. This co-supplementation reduced anxiety-like behaviors in mice and enhanced intestinal barrier function by increasing the expression of tight junction proteins [95]. Administration of Bifidobacterium breve alleviated depression and associated gastrointestinal symptoms compared to placebo in patients with MDD and reduced 5-HT turnover [96].

### 6.2. Alzheimer’s Disease and Parkinson Disease

Alzheimer’s disease (AD) and Parkinson’s disease (PD) are neurodegenerative disorders with different pathogenesis mechanisms but common features such as dysregulation of mitochondrial metabolism, oxidative stress and microglial activation, caused by tau tangles and Aβ plaques in AD and α-synuclein aggregation in PD [97]. Dysregulation of the KP has been described in the pathogenesis of both diseases, with different patterns [98]. Analysis of Trp metabolites in patients with PD revealed higher concentrations of the neuroexcitatory 3-HK and higher quinolinic acid/Kyna (QA/Kyna) ratio in plasma and cerebrospinal fluid, and lower levels of Trp, Kyn, KA, and nicotinamide in PD plasma [99]. Moreover, Heilman et al. demonstrated that high levels of 3-HK in plasma and low levels of Kyna in the cerebrospinal fluid (CSF) are associated with symptom severity and alterations in the substantia nigra pars compacta [100]. In AD, meta-analytic data indicate differences in Trp metabolite levels between cerebrospinal fluid and peripheral blood. Tryptophan levels decreased only in the peripheral blood while 3-hydroxykynurenine levels were lower in the CSF but not in the peripheral blood. Interestingly, central levels of Kyna were higher in the AD group than in the control group, while peripheral levels were lower. The kynurenine/tryptophan ratio was increased in the peripheral blood but not in the CSF. Quinolinic acid levels were unchanged in any peripheral blood compartment [101]. Van der Velpen et al. confirmed higher levels of Kyna in the CSF and higher levels of QA in AD patients than in control subjects and demonstrated that Kyna was positively associated with a lower cerebral beta-amyloid burden, while QA was associated with increased tau hyperphosphorylation and neuronal injury (tau and pTau-181) [102]. Moreover, Parker et al. demonstrated that plasma Kyn/Trp correlates over time with neurofibrillary tau pathology, neuroinflammation (GFAP), and neurodegeneration (NfL), all plasma AD biomarkers [103].

Transitioning from human biomarker studies to mechanistic interventions, animal models have provided crucial insights, albeit with the limitation that murine models of neurodegeneration often fail to capture the full timeline, genetic complexity, and slow progression of human aging and protein aggregation. For example, inhibition of IDO-1 in mouse models of Parkinson’s disease improved motor dysfunction, reduced dopaminergic neuron loss and decreased serum levels of QA [104]. Additionally, IDO-1 inhibition alleviated glial-associated neuroinflammation in the brain and reduced neuroinflammation and colonic inflammation mediated by the Toll-Like Receptor 4 (TLR4)/NF-κB pathway [104]. Importantly, the neuroprotective effects of IDO-1 inhibition were dependent on the presence of gut microbiota. When the gut microbiota was depleted using antibiotics, the therapeutic effects of the IDO-inhibitor were abolished, illustrating the essential role of gut–brain interactions in neuroinflammation and neurodegeneration [104]. In Alzheimer’s disease, astrocytic IDO expression is increased in response to amyloid-ß42 and tau oligomers. IDO inhibition restores the disruption of hippocampal glycolysis, spatial memory, and lactate production, that are vital for the regulation of neurotransmitter concentrations and bioenergetic support of neurons [105].

Complementary studies have implicated microbiota-derived indoles as functional modulators of AD pathology. Preclinical studies on AD murine models have shown that the administration of certain microbial strains (Lactobacillus reuteri, Lactobacillus delbrueckii subsp. lactis, and Streptococcus thermophilus) significantly reduced soluble Aβ42 levels in the cerebral cortex and pro-inflammatory cytokines through Trp-indole-3-lactic acid (ILA) metabolism. Indeed, direct administration of ILA and Trp reduced soluble Aβ and insoluble Aβ, inhibited neuronal degeneration, and protected cognitive functions, through the upregulation of the AhR signaling pathway [106]. Sun et al. confirmed the role of microbiota-derived indoles in alleviating Alzheimer’s disease-like pathology in APP/PS1 mouse models. Indole administration reduced aggregated Aβ and hyperphosphorylated tau levels in the brain, improved synaptic plasticity, reduced neuronal synaptic lesions and microglial activation and improved gut barrier integrity [107]. A combination of environmental enrichment training and Bifidobacterium breve significantly reduced the accumulation of hippocampal amyloid, alleviated amyloid-induced cognitive impairment, enhanced synaptic plasticity by increasing levels of Brain-Derived Neurotrophic Factor, Synaptophysin, and Fibronectin Type III Domain-Containing Protein 5, and modified the gut microbiota [108].

### 6.3. Multiple Sclerosis

Multiple sclerosis (MS) is a chronic immune-mediated neurodegenerative disorder of the central nervous system, characterized by inflammatory demyelination, axonal injury, and gliosis [109]. The pathogenesis involves an autoimmune attack on the central nervous system, where autoreactive cluster differentiation (CD) 4^+^ T cells, triggered through genetic and environmental factors, cross a compromised blood–brain barrier [109]. Recent evidence has increasingly linked the progression of MS with systemic alterations in Trp metabolism and the KP [109,110]. In toxin-induced animal models of demyelination, significant reductions in plasma KA and brain 3-HK levels have been observed [110]. During the remyelination phase, the differences in these metabolites decreased to become insignificant and none eventually, suggesting that KP flux dynamically tracks with myelin repair [110]. Furthermore, Saraste et al. demonstrated that low serum levels of 3-HK are associated with enhanced microglial activation in normal-appearing white matter and thalamus in patients with MS compared to controls [111]. In patients with active MS, levels of Kyn, QA, the Kyn/Trp ratio, and the QA/Kyna ratio are higher than those in controls, reflecting a systemic shift toward neurotoxic and pro-inflammatory branches [112]. Crucially, these metabolic changes can be reversed after pharmacological treatment; Ricci et al. analyzed plasma tryptophan metabolism in relapsing–remitting multiple sclerosis patients before and after six months of ocrelizumab (OCR) therapy, showing that OCR shifted QA and XANA toward healthy levels and restored serotonin metabolism [113]. However, in the same study, microbiota-derived indole metabolites were only partially normalized, implying persistent gut–brain axis disruption despite immunotherapy [113].

The role of microbiota indole derivatives in MS reveals a highly complex, context-dependent landscape. Ntranos et al. found that the bacterial indole metabolite indoxyl sulfate correlated with increased CSF neurofilament light chain levels, a marker of neurodegeneration, lower cortical volume on MRI, and worse cognitive scores [114]. Conversely, other indole derivatives have shown potent protective roles [115]. A recent study showed that the microbial tryptophan metabolite indole-3-carboxaldehyde (3-IAld) activates the mast cell axis, driving a tolerogenic immune response that reduces inflammation and demyelination in an MS mouse model [115]. Serum 3-IAld levels inversely correlated with disease duration in patients, highlighting gut-derived metabolites as potential modulators and therapeutic targets in MS [115]. Further complicating this paradigm, Träger et al. demonstrated that a randomized intervention with a lactobacilli-rich probiotic in MS patients increased circulating and functional Tregs, increased fecal and blood concentrations of indole-3-acetic acid, and elevated anti-inflammatory cytokine IL-10 [116]. However, Montgomery et al. showed that colonization with Lactobacillus reuteri actually exacerbated experimental autoimmune encephalomyelitis (EAE), a murine model of MS, by increasing the production of aryl hydrocarbon receptor ligands [117]. This shifted immune homeostasis toward a pro-inflammatory state with an increment of Th17 cells and reduction in regulatory T cells in both the gut and CNS [117]. High levels of Trp were necessary for this L. reuteri-dependent exacerbation, while dietary Trp limitation diminished its pro-inflammatory impact and reduced EAE severity [117]. Together, these studies reveal that microbiota-derived Trp metabolites can either exacerbate or ameliorate neuroinflammation depending on the specific bacterial species, metabolic outputs, and host immune context, underscoring that therapeutic manipulation of the gut microbiome in MS must be carefully and precisely tailored [114,115,116,117].

## 7. Tryptophan Metabolism in Autoimmune and Inflammatory Disorders

Trp and its metabolites, particularly those generated through the KP and by the gut microbiota, play important roles in the regulation of immune responses and inflammatory signaling. Through interactions with immune cells and receptors such as the aryl hydrocarbon receptor, these metabolites can influence immune tolerance, cytokine production, and the balance between pro- and anti-inflammatory pathways, being involved in the pathogenesis of several autoimmune disorders.

### 7.1. Rheumatoid Arthritis

In rheumatoid arthritis (RA), human studies have consistently linked altered circulating tryptophan (Trp) and kynurenine-pathway metabolites to disease activity, inflammatory markers, and joint damage [118]. Specifically, systemic inflammation and high levels of pro-inflammatory cytokines excessively activate indoleamine 2,3-dioxygenase (IDO), the rate-limiting enzyme responsible for degrading Trp into kynurenine (Kyn) [118]. As a result, RA patients frequently exhibit decreased serum Trp levels and an elevated Kyn/Trp ratio. These changes correlate strongly with the severity of morning stiffness, increased disease progression, and systemic immune imbalances, supporting the clinical relevance of Trp metabolism as a biomarker-rich axis in RA [118].

Mechanistic and preclinical studies suggest additional complexity within the joint microenvironment itself. For instance, tryptophan 2,3-dioxygenase 2 (TDO2)—another rate-limiting enzyme in the kynurenine pathway—is markedly upregulated in RA synovial tissues and fibroblast-like synoviocytes (FLS) [119]. The overexpression of TDO2 drives FLS hyperactivation, significantly promoting their pathogenic proliferation, migration, invasion, and secretion of inflammatory mediators [119]. Notably, pharmacological inhibition or targeted knockdown of TDO2 has been shown to reduce these aggressive cellular behaviors and ameliorate arthritis severity in animal models, demonstrating that the Trp degradation pathway directly regulates FLS-mediated synovial inflammation and cartilage destruction [119].

Beyond endogenous host metabolism, the gut microbiota significantly influences this axis by converting unabsorbed dietary Trp into various indole derivatives. These microbiota-derived metabolites can exert either anti-inflammatory or pro-inflammatory effects depending on the specific metabolite and the disease model studied [120]. For example, while structurally similar, the microbial metabolites indole-3-aldehyde (IAld) and indole-3-acetic acid (I3AA) display profoundly different bioactivities in autoimmune arthritis models [120]. IAld is capable of inhibiting macrophage-derived pro-inflammatory cytokines (such as IL-1β and IL-6) but simultaneously exhibits pathogenic pro-angiogenic and pro-osteoclastogenic properties that can contribute to bone resorption; in contrast, I3AA largely lacks these specific effects [120]. Furthermore, many of these microbial indoles act as ligands for the aryl hydrocarbon receptor (AhR) to modulate mucosal barrier integrity and systemic immune tolerance, linking gut microbial dysbiosis directly to joint inflammation [121]. Accordingly, Trp metabolism in RA should be interpreted as metabolite- and context-specific rather than uniformly protective or pathogenic.

### 7.2. Systemic Lupus Erythematosus

Serum Trp levels are decreased in SLE patients compared to controls, and metabolic profiling, including tryptophan levels, may be useful for differential diagnosis between SLE and other autoimmune diseases such as Sjögren syndrome and systemic sclerosis [122,123]. Trp availability has also been associated with developing depressive behavior and anxiety [124]. Moreover, levels of Trp, together with other essential amino acids, demonstrated exceptional accuracy in predicting lupus nephritis (LN) and distinguishing SLE from LN [125]. Human metabolomic studies have consistently demonstrated altered Trp catabolism in SLE patients, with elevated Kyn, QA and the Kyn/Trp ratio, correlating with disease activity score, severe fatigue, and joint, renal and neurological involvement [126,127]. QA in particular has also been linked to cognitive impairment [128]. Fecal metabolomic analyses further identified Trp-derived metabolites as biomarkers that distinguish SLE from healthy controls. Zhang et al. performed one of the earliest fecal metabolomic analyses, finding increased Kyna and XANA in SLE patients’ samples [129]. Yan et al. found that Trp was enriched in SLE stool samples and correlated with higher disease activity score, suggesting the role of these metabolites as a non-invasive biomarker of SLE [130].

Besides the KP, recent studies have highlighted the role of the gut microbiota–tryptophan metabolism axis in the pathogenesis of SLE. However, preclinical evidence predominantly relies on genetically engineered murine models, which provide valuable immunological insights but do not perfectly mimic the spontaneous, polygenic, and heterogeneous nature of human SLE. In this context, Choi et al. demonstrated that lupus-prone mice (LP) with gut dysbiosis displayed lower Trp and 5-HT but higher Kyn in serum and feces [131]. Fecal transfer from lupus-prone mice into germ-free mice induced autoantibodies and T follicular helper cell expansion [131]. Antibiotic treatment of LP mice reduced autoimmune activation and delayed disease onset, suggesting a causal role of dysbiosis in this model [131].

Building upon these findings, the metabolomic profiling of Brown et al. revealed distinct serum and fecal Trp metabolites between lupus-prone mice and non-autoimmune controls and that high dietary Trp caused metabolic changes to a greater extent in LP mice [132]. Moreover, the study demonstrated that gut microbiota skews host Trp catabolism toward the production of indole derivatives, while reducing the KP. These microbial products, in particular tryptamine, exerted direct immunometabolic effects on CD4^+^ T cells, promoting glycolysis, thereby enhancing effector T-cell differentiation and cytokine production [132]. Some studies suggest that modulating the gut microbiota–Trp–AHR axis may have therapeutic benefits in SLE. Pan et al. [133] showed that transplantation of human umbilical cord-derived mesenchymal stem cells (hUC-MSC) in lupus-prone mice reduced autoantibody production and inflammatory response, ameliorating gut microbiota dysbiosis and increasing beneficial metabolites such as 3-indoleacrylic acid, 1-acetylindole, and tributyrin, which ameliorate intestinal damage via the AhR. A study by Wu et al. demonstrated that Bifidobacterium supplementation, along with its metabolites and short-chain fatty acids, in lupus prone mice, corrected imbalances in CD4^+^ T and B cell subsets, reduced autoantibody titers and ameliorated glomerular injury [134].

### 7.3. Psoriasis

In psoriasis, an immune-mediated chronic inflammatory skin disease characterized by hyperproliferation of keratinocytes and infiltration of immune cells, both the host kynurenine pathway and microbiota-related Trp metabolites have been heavily implicated in disease biology [135,136]. Human metabolomic studies report significantly altered peripheral kynurenine pathway metabolites in patients with psoriasis, including disrupted Kyna and quinolinic acid profiles that correlate with systemic inflammatory burden [136]. Mechanistic studies suggest that specific enzymes within this pathway act to amplify inflammatory signaling within psoriatic lesions [137,138]. For example, L-kynureninase has been identified as a novel inflammatory factor in psoriasis, and its overexpression contributes directly to pathogenesis through pro-inflammatory effects in the skin [137,138]. The role of indoleamine 2,3-dioxygenase (IDO) isoforms in psoriasis is multifaceted and highlights the regulatory complexity of Trp degradation. While some studies report that the loss of IDO1 does not significantly affect the severity of skin lesions in certain psoriasis models [139], others demonstrate that IDO is essential for mediating the immunosuppressive effects of mesenchymal stromal cells on hyperactive T cells [140]. Furthermore, IDO2 deficiency has been shown to exacerbate imiquimod-induced psoriasis-like skin inflammation, suggesting that baseline Trp catabolism may serve as an essential negative feedback loop to keep skin inflammation in check [141].

Beyond endogenous host metabolism, the gut-skin axis and microbial Trp metabolism are emerging as critical regulators of psoriatic inflammation [142]. Selected microbial or skin-derived indole metabolites function as crucial signaling molecules that can activate AhR-dependent anti-inflammatory pathways and improve barrier-relevant signaling in experimental models [143,144]. For instance, indole-3-lactic acid (ILA) has been shown to inhibit keratinocyte hyperproliferation through AhR activation [144], while quinolinic acid can negatively regulate the NLRP3 inflammasome via the AhR in psoriatic environments [143]. The composition of the gut microbiome directly dictates the availability of these protective metabolites; recent studies indicate that gut microbe-derived metabolites drive psoriatic inflammation largely via the downstream modulation of skin-homing Th17 cells [142]. Consequently, pharmacological or topical interventions that target this metabolic axis hold significant translational promise. Metformin treatment, for example, has been shown to protect mice from psoriasis-like symptoms specifically through the modulation of bacterial tryptophan metabolites [145], and topical application of magnolol ameliorates dermatitis by regulating local Trp metabolism [146]. As in other immune-mediated conditions, the available evidence supports the biological relevance of Trp metabolism in psoriasis but still requires careful separation of human biomarker associations from preclinical therapeutic promise [135,136,137,138,139,140,141,142,143,144,145,146].

## 8. Discussion

While the individual studies reviewed herein highlight the critical involvement of Trp metabolism across diverse pathologies, a cross-disease synthesis reveals both consistent metabolic patterns and notable contradictions. A recurring hallmark across digestive (e.g., IBD, CRC), cardiometabolic, autoimmune, and neurodegenerative disorders is the inflammation-driven shunting of Trp toward the kynurenine pathway. This systemic shift, typically driven by cytokine-mediated upregulation of IDO1 or TDO, frequently correlates with increased disease severity and immune dysregulation. However, significant disease-specific differences exist. For example, while the microbial production of indole derivatives generally confers barrier-protective and anti-inflammatory effects in intestinal (IBD) and metabolic (T2D) contexts, these same metabolites can drive context-dependent pathogenic responses, such as exacerbated neuroinflammation in multiple sclerosis or altered osteoclastogenesis in rheumatoid arthritis.

Understanding these contradictions requires careful attention to critical confounding factors that are frequently underreported or highly variable across studies. The interpretation of the Kyn/Trp ratio and absolute metabolite concentrations must account for host age, sex, and baseline dietary Trp intake. Furthermore, systemic levels are heavily influenced by concurrent medications, acute versus chronic disease activity, and underlying renal or hepatic function, which directly dictate the clearance or accumulation of metabolites like indoxyl sulfate and QA. Methodological heterogeneity also limits cross-study comparability, as variations in metabolomic platforms, sample handling, and differences in baseline microbiota composition heavily influence the detected metabolic signatures.

Crucially, the current literature often struggles to definitively separate association from causation. Alterations in Trp metabolism observed in cross-sectional human cohorts may merely reflect the secondary byproducts of a dysbiotic and inflammatory environment rather than serving as the primary drivers of disease. Consequently, while therapeutic strategies such as dietary Trp supplementation, probiotic administration, microbial metabolite delivery, AhR ligands, and targeted enzymatic inhibitors show immense promise, they are largely based on experimental and preclinical models. Translating these interventions to clinical practice requires addressing complex pharmacological challenges. Future clinical trials must rigorously evaluate safety, dose dependency, and tissue specificity, acknowledging the pleiotropic nature of these pathways. As demonstrated by the dual roles of various metabolites, therapeutic efficacy is not simply a matter of presence or absence; rather, the same metabolite may exert protective or pathogenic effects highly dependent on its local concentration, the specific receptors activated (e.g., varying AhR binding affinities), the baseline microbial context, and the stage of the disease.

## 9. Conclusions and Future Perspectives

The understanding of tryptophan (Trp) metabolism has expanded considerably, revealing its fundamental role not merely as a collection of isolated biochemical pathways, but as a crucial mechanistic bridge connecting nutrition, the gut microbiota, the host immune response, and the development of chronic diseases. Dictated by dietary intake and subsequently partitioned by complex host and microbial enzymatic networks, Trp acts as a molecular sensor that translates environmental and nutritional inputs into profound systemic consequences. Across digestive, cardiometabolic, neuropsychiatric, and autoimmune disorders, several recurring pathological patterns emerge. Chronic inflammatory states consistently shift this balance toward kynurenine-pathway activation, driving metabolic dysfunction and systemic immune dysregulation. Conversely, specific microbial indole derivatives frequently exhibit barrier-protective and immunoregulatory properties, while serotonin-related alterations disrupt gastrointestinal motility and gut–brain communication.

By acting as this integrative bridge, Trp metabolism provides a powerful framework for deciphering the multifactorial pathogenesis of chronic diseases. These observations strongly support the translational value of Trp-derived metabolites as candidate biomarkers for disease phenotyping and clinical stratification. However, they also highlight the absolute necessity for careful, context- and disease-specific interpretation. It is essential to explicitly acknowledge the inherent limitations of this narrative-review methodology, which include possible selection bias, the profound heterogeneity of analytical methods used across studies, inconsistent biological matrices, and the absence of formal evidence grading. Consequently, any recommendations concerning biomarkers or therapeutic modulation discussed herein must remain strictly disease-specific and should not imply immediate clinical applicability. Future research must prioritize longitudinal human cohorts, standardized metabolite reporting, and the integrated analysis of diet, microbiota composition, host inflammation, and treatment responses. Crucially, future investigations must also clearly differentiate between biological compartments, recognizing that systemic measurements in serum or plasma cannot be directly interchanged with localized concentrations in feces, target tissues, or cerebrospinal fluid.

## Figures and Tables

**Table 1:** Major pathways of tryptophan metabolism.

Pathway	Key Enzymes	Major Metabolites	Principal Tissues/Cellular Sources	Main Physiological Functions	Representative Disease Associations
Kynurenine pathway	TDO, IDO1/IDO2, KMO, KATs	Kynurenine, kynurenic acid, 3-hydroxykynurenine, quinolinic acid, xanthurenic acid	Liver; peripheral tissues; immune cells including dendritic cells and other inflammatory cell populations	Major route of host Trp degradation; immune regulation; inflammation-responsive metabolic rerouting; neuroactive metabolite generation; contribution to NAD+ biosynthesis	IBD, celiac disease, gastrointestinal cancers, MASLD/MASH, T2D, CVD, CKD, depression/anxiety, AD, PD, MS, RA, SLE, psoriasis
Serotonin pathway	TPH1, TPH2, aromatic amino acid decarboxylase, MAO-A	5-hydroxytryptophan, serotonin (5-HT), 5-HIAA	Enterochromaffin cells in the gut; raphe neurons in the CNS; platelets as a storage pool	Regulation of gut motility, secretion, visceral sensation, gut–brain signaling, mood, appetite, sleep, cognition	IBS, IBD, gastroparesis, SIBO, depression/anxiety, gut–brain interaction disorders
Melatonin pathway	AANAT, ASMT/HIOMT	N-acetylserotonin, melatonin	Primarily pineal gland; also extra-pineal production discussed more broadly in physiology contexts	Circadian regulation; sleep–wake signaling; antioxidant and cytoprotective effects	Disorders involving circadian disruption, oxidative stress, and neuroimmune imbalance; potentially relevant to neurological/psychiatric disease contexts
Microbial indole pathway	Bacterial tryptophanase (TnaA), indolelactate dehydrogenase, reductive bacterial enzymes, decarboxylation pathways	Indole, indole-3-propionic acid, indole-3-lactic acid, indole-3-acetic acid, tryptamine	Gut microbiota, particularly distal intestinal commensals; cross-feeding microbial networks	Maintenance of epithelial barrier integrity; AhR signaling; mucosal immune regulation; antimicrobial defense; modulation of motility and host serotonin signaling; gut–organ communication	IBD, IBS, celiac disease, MASLD, T2D, CKD/CVD, depression/anxiety, AD, MS, RA, SLE, psoriasis

Abbreviations: AANAT, arylalkylamine N-acetyltransferase; AD, Alzheimer’s disease; AhR, aryl hydrocarbon receptor; ASMT/HIOMT, acetylserotonin O-methyltransferase/hydroxyindole-O-methyltransferase; CKD, chronic kidney disease; CNS, central nervous system; CVD, cardiovascular disease; IBD, inflammatory bowel disease; IBS, irritable bowel syndrome; IDO1/IDO2, indoleamine 2,3-dioxygenase 1/2; KATs, kynurenine aminotransferases; KMO, kynurenine 3-monooxygenase; MASH, metabolic dysfunction-associated steatohepatitis; MASLD, metabolic dysfunction-associated steatotic liver disease; MS, multiple sclerosis; NAD^+^, nicotinamide adenine dinucleotide; PD, Parkinson disease; RA, rheumatoid arthritis; SERT, serotonin reuptake transporter; SIBO, small intestinal bacterial overgrowth; SLE, systemic lupus erythematosus; T2D, type 2 diabetes mellitus; TDO, tryptophan 2,3-dioxygenase; TnaA, bacterial tryptophanase; TPH1/TPH2, tryptophan hydroxylase 1/2; Trp, tryptophan; 5-HIAA, 5-hydroxyindoleacetic acid; 5-HT, 5-hydroxytryptamine (serotonin).

**Table 2:** Digestive disorders and tryptophan metabolism.

Disease	Dominant Pathway Alteration	Key Metabolites/Markers	Main Evidence Type (Human/Preclinical/Both)	Putative Clinical Implications
Inflammatory bowel disease (IBD)	Increased kynurenine-pathway activation; reduced protective microbial indole signaling; altered Trp transport	Lower circulating Trp, higher fecal Trp, elevated Kyn/Trp ratio, altered kynurenine metabolites, reduced IPA/IAA-related signaling	Both	Biomarker potential for inflammatory activity; mechanistic relevance to barrier dysfunction and mucosal immune imbalance; possible future pathway-targeted therapies
Irritable bowel syndrome (IBS)	Altered serotonin-pathway activity and microbiota-linked Trp metabolism; possible subtype-specific shifts toward serotonin vs. kynurenine activity	5-HIAA, serum serotonin, tryptamine, fecal Trp, kynurenine-related metabolites	Mainly human, with mechanistic support	May help explain symptom subtype, motility changes, visceral sensitivity, and gut–brain symptoms; not yet an established intervention pathway
Celiac disease	Inflammatory kynurenine shunting; altered AhR-ligand generation by microbiota	Reduced plasma Trp, elevated Kyn/Trp ratio, increased IDO expression	Both, but therapeutic implications mainly preclinical/mechanistic	Supports biological role of Trp metabolism in mucosal immune regulation; clinical therapeutic application remains limited
Gastrointestinal cancers (especially CRC)	Strong kynurenine-pathway activation in tumor microenvironment	IDO1 overexpression, local Trp depletion, kynurenine accumulation, AhR activation	Both, with strong mechanistic emphasis	Possible prognostic/metabolic biomarker role; rationale for immunometabolic therapeutic targeting
Gastroparesis	Functional relevance of impaired serotonin signaling secondary to reduced Trp availability or altered 5-HT biology	5-HT pathway markers conceptually relevant rather than well-established disease biomarkers	Limited human plus mechanistic/experimental	Biological plausibility for motility-related relevance; evidence still limited compared with IBD/IBS
Small intestinal bacterial overgrowth (SIBO)	Altered small-intestinal bacterial handling of Trp with serotonin-pathway dysregulation	Urinary 5-HIAA, serotonin-pathway signatures	Limited human	Preliminary biomarker and phenotype-stratification potential; evidence base still early
Liver diseases/MASLD-MASH/cirrhosis/hepatic encephalopathy	Opposing pathway effects: gut-derived serotonin may promote steatosis; kynurenine-pathway activation rises with inflammatory progression; neuroactive metabolites may accumulate in advanced disease	Kyn/Trp ratio, quinolinic acid-related neuroactive metabolites, serotonin-related hepatic signaling	Both	Stage-specific biomarker and mechanistic relevance across gut–liver axis disorders; possible pathway-specific therapeutic targeting

Abbreviations: AhR, aryl hydrocarbon receptor; CRC, colorectal cancer; IAA, indole-3-acetic acid; IBD, inflammatory bowel disease; IBS, irritable bowel syndrome; IDO1, indoleamine 2,3-dioxygenase 1; IPA, indole-3-propionic acid; Kyn, kynurenine; Kyn/Trp ratio, kynurenine-to-tryptophan ratio; MASH, metabolic dysfunction-associated steatohepatitis; MASLD, metabolic dysfunction-associated steatotic liver disease; SIBO, small intestinal bacterial overgrowth; Trp, tryptophan; 5-HIAA, 5-hydroxyindoleacetic acid; 5-HT, serotonin.

**Table 3:** Extra-digestive/systemic disorders and tryptophan metabolism.

Disease	Dominant Pathway Alteration	Key Metabolites/Markers	Main Evidence Type (Human/Preclinical/Both)	Putative Clinical Implications
Type 2 diabetes mellitus	Increased kynurenine-pathway activity; reduced protective microbial indole signaling	Kynurenine, xanthurenic acid, quinolinate, Kyn/Trp ratio, indolepropionate	Mainly human, supported by mechanistic data	Risk stratification, metabolic biomarker development, and pathway-specific prevention concepts
Metabolic dysfunction-associated steatotic liver disease (MASLD)	Gut-derived serotonin may be pathogenic; microbial indole metabolites may be protective	Serotonin-related hepatic signaling, indole, indolepropionate	Both	Supports pathway-specific interpretation of Trp metabolism rather than a single overall readout
Cardiovascular disease (CVD)	Inflammatory kynurenine-pathway activation associated with vascular risk	Kyn/Trp ratio, circulating Trp, indoxyl sulfate, indolepropionate	Mainly human observational plus mechanistic support	Cardiovascular risk biomarker potential; links inflammation and microbial metabolism to vascular biology
Chronic kidney disease (CKD)	Accumulation of host-derived and microbial Trp metabolites because of reduced renal clearance	Indoxyl sulfate, kynurenine-pathway metabolites, albumin-bound indolic toxins	Both	Strong biomarker and mechanistic relevance for vascular complications and uremic toxicity
Depression and anxiety	Increased kynurenine-pathway imbalance with altered serotonergic and microbiota-linked signaling	Lower Trp, lower kynurenic acid in some settings, higher Kyn/Trp ratio, 3-HK-related shifts	Both	Supports biomarker exploration and gut–brain mechanistic interpretation, but causality remains difficult to define in humans
Alzheimer’s disease	Kynurenine-pathway imbalance with compartment-specific central vs. peripheral metabolite changes; microbial indole effects mainly preclinical	Kyn/Trp ratio, kynurenic acid, quinolinic acid, AD biomarker correlations	Both	Candidate biomarker pathway and experimental therapeutic target, especially for neuroinflammation and neurodegeneration
Parkinson disease	Shift toward neuroexcitatory kynurenine metabolites and reduced protective metabolites	3-HK, QA/KA ratio, lower Trp, lower kynurenic acid/nicotinamide	Both	Potential biomarker pathway linked to disease severity and neuroinflammation
Multiple sclerosis	Increased kynurenine-pathway activation; microbiota-derived indole effects may be protective or pathogenic depending on context	Kyn, QA, Kyn/Trp ratio, QA/KA ratio, indoxyl sulfate, indole-3-carboxaldehyde	Both	Strong biological relevance, but therapeutic extrapolation must be cautious because effects are context-dependent
Rheumatoid arthritis	Altered circulating kynurenine-pathway profile associated with disease activity; mixed effects of microbial indoles	Kynurenine, kynurenic acid, xanthurenic acid, QA-related profiles	Both	Biomarker-rich axis for disease activity and immune regulation; metabolite-specific therapeutic interpretation needed
Systemic lupus erythematosus (SLE)	Reduced Trp with increased kynurenine-pathway activation; microbiota-driven Trp metabolism implicated in immune activation	Trp, Kyn, QA, Kyn/Trp ratio, fecal Trp-derived metabolites	Both, but interventional evidence mostly preclinical	Potential for biomarker development in disease activity and lupus nephritis; therapeutic field still emerging
Psoriasis	Dysregulated kynurenine-pathway signaling plus possible protective AhR-mediated indole effects	Kynurenic acid, quinolinic acid-related profiles, indole/AhR-related metabolites	Both	Supports disease relevance of Trp metabolism, but translational claims remain ahead of clinical validation

Abbreviations: AD, Alzheimer’s disease; CKD, chronic kidney disease; CVD, cardiovascular disease; KA, kynurenic acid; Kyn, kynurenine; Kyn/Trp ratio, kynurenine-to-tryptophan ratio; MASLD, metabolic dysfunction-associated steatotic liver disease; MS, multiple sclerosis; PD, Parkinson disease; QA, quinolinic acid; QA/KA ratio, quinolinic acid-to-kynurenic acid ratio; RA, rheumatoid arthritis; SLE, systemic lupus erythematosus; T2D, type 2 diabetes mellitus; Trp, tryptophan; 3-HK, 3-hydroxykynurenine.

## Data Availability

No new data were created or analyzed in this study. Data sharing is not applicable to this article.

## Abbreviations

The following abbreviations are used in this manuscript: **5-HIAA**: 5-hydroxyindoleacetic acid, **5-HT**: serotonin, **5-HTP**: 5-hydroxytryptophan, **3-IAld**: indole-3-carboxaldehyde, **ACE2**: angiotensin-converting enzyme II, **AD**: Alzheimer’s disease, **AhR**: aryl hydrocarbon receptor, **AIDS**: acquired immunodeficiency syndrome, **CD**: Crohn’s disease, **CeD**: celiac disease, **CIA**: collagen-induced arthritis, **CKD**: chronic kidney disease, **CNS**: central nervous system, **CRC**: colorectal cancer, **CVD**: cardiovascular disease, **DCs**: dendritic cells, **DSS**: dextran sodium sulfate, **EC cells**: enterochromaffin cells, **ENS**: enteric nervous system, **GI**: gastrointestinal, **GLP-1**: glucagon-like peptide-1, **hUC-MSC**: human umbilical cord-derived mesenchymal stem cells, **HE**: hepatic encephalopathy, **IBD**: inflammatory bowel disease, **IBS**: irritable bowel syndrome, **IBS-C**: irritable bowel syndrome with constipation, **IBS-D**: irritable bowel syndrome with diarrhea, **IDO**: indoleamine 2,3-dioxygenase, **IDO1**: indoleamine 2,3-dioxygenase 1, **IEC**: intestinal epithelial cells, **IEL**: intraepithelial lymphocytes, **IFN-γ**: interferon-gamma, **IL** interleukin, **ILA**: indole-3-lactic acid, **IPA**: indole-3-propionic acid, **KATs**: kynurenine aminotransferases, **KP**: kynurenine pathway, **Kyn/Trp**: kynurenine-to-tryptophan ratio, **Kyna**: kynurenic acid, **LAT1**: large neutral amino acid transporter 1, **LILP**: large intestine lamina propria, **LPS**: lipopolysaccharide, **LP**: lupus-prone mice, **MASLD**: metabolic dysfunction-associated steatotic liver disease, **MDD**: major depressive disorder, **MS**: multiple sclerosis, **NAFLD**: non-alcoholic fatty liver disease, **NASH**: non-alcoholic steatohepatitis, **NF-κB**: nuclear factor kappa-light-chain-enhancer of activated B cells, **NLRP3**: NLR family pyrin domain containing 3, **NMDA**: N-methyl-D-aspartate, **OCR**: ocrelizumab, **PD**: Parkinson’s disease, **PD-L1**: programmed death-ligand 1, **QA** quinolinic acid, **QUIN**: quinolinic acid, **RA**: rheumatoid arthritis, **RALDH2**: retinaldehyde dehydrogenase 2, **Ret**: retinol, **SERT**: serotonin reuptake transporter, **SIBO**: small intestinal bacterial overgrowth, **SLE**: systemic lupus erythematosus, **SNPs**: single-nucleotide polymorphisms, **T1D**: type 1 diabetes, **T2D**: type 2 diabetes, **TAT1**: T-type amino acid transporter 1, **TDO**: tryptophan 2,3-dioxygenase, **TDO2**: tryptophan 2,3-dioxygenase 2, **TGF-β**: transforming growth factor beta, **TLR4**: Toll-like receptor 4, **TNF-α**: tumor necrosis factor alpha, **TnaA**: tryptophanase, **TPH1**: tryptophan hydroxylase 1, **TPH2**: tryptophan hydroxylase 2, **Trp**: tryptophan, **Treg**: regulatory T cells, **TME**: tumor microenvironment, **UC**: ulcerative colitis, **WHO**: World Health Organization, **XANA**: xanthurenic acid.
